# The meso-connectomes of mouse, marmoset, and macaque: network organization and the emergence of higher cognition

**DOI:** 10.1093/cercor/bhae174

**Published:** 2024-05-20

**Authors:** Loïc Magrou, Mary Kate P Joyce, Sean Froudist-Walsh, Dibyadeep Datta, Xiao-Jing Wang, Julio Martinez-Trujillo, Amy F T Arnsten

**Affiliations:** Department of Neural Science, New York University, New York, NY 10003, United States; Department of Neuroscience, Yale University School of Medicine, New Haven, CT 06510, United States; School of Engineering Mathematics and Technology, University of Bristol, Bristol, BS8 1QU, United Kingdom; Department of Psychiatry, Yale University School of Medicine, New Haven, CT 06510, United States; Department of Neural Science, New York University, New York, NY 10003, United States; Departments of Physiology and Pharmacology, and Psychiatry, Schulich School of Medicine and Dentistry, Western University, London, ON, N6A 3K7, Canada; Department of Neuroscience, Yale University School of Medicine, New Haven, CT 06510, United States

**Keywords:** cortex, hippocampus, primate, rodent, working memory

## Abstract

The recent publications of the inter-areal connectomes for mouse, marmoset, and macaque cortex have allowed deeper comparisons across rodent vs. primate cortical organization. In general, these show that the mouse has very widespread, “all-to-all” inter-areal connectivity (i.e. a “highly dense” connectome in a graph theoretical framework), while primates have a more modular organization. In this review, we highlight the relevance of these differences to function, including the example of primary visual cortex (V1) which, in the mouse, is interconnected with all other areas, therefore including other primary sensory and frontal areas. We argue that this dense inter-areal connectivity benefits multimodal associations, at the cost of reduced functional segregation. Conversely, primates have expanded cortices with a modular connectivity structure, where V1 is almost exclusively interconnected with other visual cortices, themselves organized in relatively segregated streams, and hierarchically higher cortical areas such as prefrontal cortex provide top–down regulation for specifying precise information for working memory storage and manipulation. Increased complexity in cytoarchitecture, connectivity, dendritic spine density, and receptor expression additionally reveal a sharper hierarchical organization in primate cortex. Together, we argue that these primate specializations permit separable deconstruction and selective reconstruction of representations, which is essential to higher cognition.

## Introduction

The underlying architecture of the mammalian cortex is crucial to understanding the neural bases of cognition, and comparative studies allow insight into mechanisms of brain evolution and species-specific specializations. Investigations into inter-areal connectivity, now known as the mesoscale connectome (Zeng 2018), predate the connectomics era by several decades and are tightly linked to the technical advances of tract-tracing (TT; see Cowan 1998; Lanciego and Wouterlood 2020). As was often the case in neuroscience, much of the effort started with the macaque visual system, including prefrontal cortical (PFC) visual association areas, such as the frontal eye fields (FEFs) and area 46. The 1980s were witness to many fundamental publications exploring many pathways and connections, eventually to be brought together in Felleman and Van Essen (1991), where it was for the first time presented in a single table of inter-areal connectivity. The terminology used back then is revealing: Although it very much was the matrix of a network, it was just described as a table, thus retrospectively showing the difference between this seminal work and our current connectome concept. In many ways, connectomics is anatomy combined with graph theory. The concept of connectome itself, a term independently coined by Sporns et al. (2005) and Hagmann (2005), had to wait 14 more years and can be summarized as follows: *a network-based approach to understanding the brain’s connectivity at all scales, from structure to function*. This importantly differs from the simpler study of connectivity in both scope and method, aspiring to integrate micro-, meso-, and macroscales together, which is to say understanding their interactions, through the lens of network science and graph theory. As the terms of graph theory may be unknown to many readers, a guide is provided in Box 1.

**Box 1.** A brief guide to graph theory terminology.

***Graph*.** A graph is an abstract representation of a network. They are composed of only two elements: nodes (also called vertices) and links (also called edges). A graph can be directed, meaning that the links have directionality (represented by arrows), where a link going from A to B (A → B) is different from a link going from B to A (B → A), or undirected (represented by simple lines), where A → B is identical to B → A. A graph can also be weighted, meaning that a weight is attributed to each link, representing their magnitude; or binary (i.e. nonweighted) where a link simply exists or does not exist. These two categories of graphs are orthogonal to each other and nonmutually exclusive.

***Adjacency matrix.*** Although graphs are often graphically represented with points and lines (or arrows), they are usually thought about and handled as matrices. The adjacency matrix of a graph is a matrix where each row and column is a node of the graph, and every entry in the matrix represents a link. A binary matrix will only be filled with 0s and 1s, when a weighted matrix can hold any values, with no bound necessary. An undirected graph will always have a symmetrical matrix, because A → B is identical to B → A, but a directed graph can have an asymmetrical matrix.

***Density.*** The density of a graph measures its completeness. A complete graph means that the matrix is full, and therefore, all possible connections actually exist. On the opposite side, an empty graph means the matrix is empty. Density itself is the number of links that do exist divided by the number of links that could, diagonal excluded. If the graph is complete, the density is 1 and falls to 0 if the graph is empty. A graph is said to be dense if its density is close to reaching maximal value, and said to be sparse when density is close to 0.

***Degree.*** The *degree* of a node (or vertex) is simply the number of links attached to said node. In a directed graph, the notion should be divided into two: *in-degree* and *out-degree*. The in-degree of a node is, quite transparently, the number of in-coming links and the out-degree the number of out-going links. High degree nodes will, in some topologies, be called hubs.

***Average shortest path length.*** A crucial notion for networks, especially if one is interested in efficiency of information passing. Length here should be understood to mean topological distances, that is, the number of node-to-node jumps one needs to make in order to get from an arbitrary starting node to any target node. For any pair of nodes, there is usually a diversity of paths (i.e. sequence of links) that are linking the two nodes, ranging from the shortest possible to potentially infinitely long and convoluted paths. Taking the shortest possible path gives information on how topologically close the pair of nodes is. If one does that for every possible pair of nodes, one can then compute the average, thus giving information about the network as a whole. A graph with low average shortest path length means that nodes are on average close to each other within that particular network. In other words, getting from any point to any other point in the network takes a small number of “hops,” which is a good way to ensure good flow of information.

***Clustering coefficient.*** As for node degree, the clustering coefficient is computed for each node, and can then be averaged over all nodes as a global property. It essentially asks the question: *Are my friends friends with each other?* For any given node, one takes the set of direct neighbors (i.e. nodes directly connected to the node in question) and counts the number of links that do exist in that set, divided by the number of links that could exist (also in that set). It is basically a measure of the density, computed for the subnetwork defined by the direct neighbors of a node. Said differently, it quantifies how close the neighbors are from being a *clique*, which is itself defined as a subset of nodes within the graph that reaches a density of 1.

***Modularity.*** Related to clustering and yet different, modularity is a global metric that measures the existence of a *community structure* in the network. In other words, modularity quantifies how easy it is to separate a graph into different modules/communities. A community can be defined as a subset of nodes that are more densely connected together than they are with the outside of that subset. Said differently, two nodes are more likely to be connected if they belong to the same community. There are in fact many ways to detect communities in a network, and a modularity value cannot be computed without having assigned all nodes to at least one community. Once that is done by whatever means, the number of edges within a community is divided by the total number of existing edges in the graph (i.e. the fraction of in-module links) and is compared by subtraction to the expected fraction that would occur in a randomized version of the same graph (i.e. with equal number of nodes and links), usually preserving the degree of each node.

***Dyadic and triadic motifs.*** Those small subsets of nodes of size 2 or 3 that repeat themselves throughout the network. In a way, they are the fundamental building blocks of every network. A motif is a specific pattern of links in a given subgraph. Counting the occurrences of each motif is called a census, and the number of different motifs depends on the number of nodes one chooses for the breakdown. For dyads (2-node motifs), there are only 3 of them: empty, unidirectional, and bidirectional. Hence, a dyad census in a directed graph is just counting empty links, 1-sided arrows, and 2-sided arrows. On the other hand, a triad (3-node motif) is best understood as a triangle of nodes, where each side of the triangle (i.e. links) can be empty, unidirectional, or bidirectional. The number of possible combinations is now 16, meaning that a triad census already has 16 different motifs ranging from empty to fully connected bidirectionally and all possible combinations in between. The distribution of those motifs can be characteristic to the family of graphs the network belongs to and is often described as the fingerprint of a network. It does not, however, necessarily relate to the function of the network.

***Topology.*** In general, topology is the study of object properties that are not modified under continuous geometrical deformations, such as bending, stretching, and twisting. In the case of networks, networks organized as an “S,” an “L,” or a “U” are topologically identical, but different from an “O,” and itself different from a “B.” In the realm of real-world networks, topology is a word used with an added level of abstraction, and several types of topologies can be used to describe a single network. A random graph can also be small-world (see below), and although two separate random graphs have only a very slim chance of being topologically identical, their randomness does capture something important about their topology (and so would their small-wordness). Closer to connectomics, an important type of topology, passionately debated in the field, is that of *small-world* topology, evoked just above. A small-world network is defined by a high clustering coefficient (higher than its randomized equivalent), and a small average shortest path length (similar to its randomized equivalent, random graphs usually having very short path length compared to their size). In other words, most of one’s friends are also friends together, and one can go to any other point in the network in only a few hops. Small-world properties are thought to be very common in many real-world networks, from the web to gene networks. One of their characteristics is the existence of *hub* nodes, with a relatively higher degree than others, which link together the different clusters.

The interareal cortical connectomes of the mouse, marmoset and macaque are becoming increasingly realized (Markov et al. 2014a; Rosa et al. 2014; Gămănuţ et al. 2018), offering the opportunity for rich comparisons across species. These analyses have confirmed the general tenet that smaller brains—e.g. in rodents—have greater widespread, all-to-all “dense” interareal connectivity compared to larger brains, due to volume constraints imposed by the skull, and that long-range connections in particular are more sparse in larger primate brains, with cortical regions thus more specialized (Horvát et al. 2016). This pattern in mammalian brains is consistent with wider principles, i.e. that smaller nervous systems necessarily perform multisensory level processing at earlier stages in sensory cortical streams, and that smaller nervous systems are more interconnected, arguably even at the synaptic level. In drosophila, a single presynaptic terminal (a “T-bar”) makes synaptic contact with many dendrites (Scheffer 2020), whereas in primate association cortex, most cortico-cortical connections are a single input onto a single spine (Peters et al. 1991; Cano-Astorga et al. 2021). Analyses of the long-range connections in macaque, and their presumed extrapolations to the human cortex, have led to speculations that this organization makes large-brained primates more vulnerable to focal injury (e.g. stroke) due to reduced redundancy. This topological organization may be more conducive to disconnection syndromes like aphasia, but may also allow for the rise of higher cognition (Ringo 1991; Kaas 2000; Horvát et al. 2016; Goulas et al. 2019; Wang et al. 2020; Ardesch et al. 2022).

In 2021, Changeux and colleagues (Changeux et al. 2021) produced an outstanding review exploring the link between mammalian brain size and cognitive abilities of the human brain, including language, and the genetic specificities behind it. They argue that increase in connectome modularity is a key aspect of brain expansion that allows for complex cognition to happen, such as working memory. Far from going against their claims, the current review explores similar questions from the other end of the mammalian brain continuum, by highlighting the recent findings that have emerged from comparisons of the mouse, marmoset, and macaque connectomes, and explores how these major differences in connectivity patterns may influence the neural representations underlying cognition. We suggest that a highly dense connectome, as seen in the small mouse brain, not only provides robustness against lesions (i.e. through redundancies) but also likely facilitates multimodal integrative processes at earlier sensory stages, in the service of rapid motor and emotional response to environmental threats. However, dense connectivity may limit the power of abstract cognitive tasks, where information held in working memory must be precisely specified, dissociated, and reconstructed based on relevance to top–down goals. We explore how a more modular network structure may be essential for abstraction and higher cognition, providing a cache of “raw data” in early sensory cortices that can be utilized in multiple ways. However, this requires a large cortex that can process multiple, effectively segregated domains and with sufficient top–down control to flexibly organize information as context demands. The following reviews some of the anatomical, physiological, and molecular data to support these ideas.

## Summary of areal connectivity data

### From mouse to marmoset to macaque, three connectomes, one principle

The mesoscale connectome, investigated by means of retrograde TT, reveals several key organizing principles that appear to hold across the species so far studied (Markov et al. 2014a; Gămănuţ et al. 2018; Theodoni et al. 2021). The first principle is that of network density (see *density*, Box 1), where there are striking differences between primate and mouse cortex. A density of 1 indicates that any area is connected with all other areas, while a density of 0 indicates that any area is connected to no other areas. Estimates of density have varied based on the use of different TT and sampling techniques; thus, it is critically important when comparing across species to compare studies that used the same paradigm to capture connectivity. In the macaque, original estimates varied quite a bit, ranging from 0.05 to 0.58 (Markov et al. 2013b), due to differences in TT methods. However, the methodologically consistent, high sampling frequency data of the Kennedy/Knoblauch group yields a density of 0.66 (Markov et al. 2014a), now marginally lowered to 0.62 with the addition of 11 injections (Froudist-Walsh et al. 2021).

This notion of density, although simplistic because unweighted and binary, heavily influences the possible topologies that the cortical network can take (see *topology*, Box 1). Specifically, such a high inter-areal density forbids certain families of topologies that were argued for at the time, most notably that the cortex could be of a small-world topology (Tononi et al. 1994; Sporns et al. 2000; Sporns and Honey 2006; Honey et al. 2007). As explained in Box 1, a small-world network is defined by high clustering/small average shortest path length compared to a randomized version of the same graph. At 62% density, however, the randomized rewiring of the graph cannot possibly reduce the clustering coefficient, and the small-world definition fails to be efficiently applied (For a detailed review on this issue, see Markov et al. 2013a; Knoblauch et al. 2016).

Turning now to the mouse, two studies, published in parallel in 2014, proposed two independent datasets for inter-areal connectivity, one using a viral anterograde tracer (Oh et al. 2014) and the other combining anterograde and retrograde tracing (Zingg et al. 2014). Taken together, along with later re-evaluation (Horvát et al. 2016; Ypma and Bullmore 2016), those studies offered density values ranging from 0.32 to 0.73. As with the primate studies, this vast range in density values may seem confusing, but is due to the differences in methods used to analyze the TT data. Thus, it is critical to use the same methodological paradigm when comparing across species. In 2018, Gămănuţ and colleagues (Gămănuţ et al. 2018) published high-sampling-frequency retrograde TT data in the mouse, using the same paradigm as used in the macaque (Markov et al. 2014a), and thus allowing for direct comparisons across species. Using the same methods as that in macaque, they showed that mouse cortex had a density of 0.97, consistent with prior, more localized figures (Wang et al. 2012). In other words, in the mouse, apart from a few missing connections, any (cortical) area is directly connected to any other area.

Recently, the Rosa group produced a retrograde TT, marmoset connectivity matrix, with a methodology equivalent in dye and sampling frequency to the Kennedy/Knoblauch group (Theodoni et al. 2021). Their results yield a density of 0.62, very much on par with the macaque. The differences in parcellation schemes (117 for the marmoset against 91 for the macaque), goes in the direction of the macaque having a slightly sparser connectivity if it had an equally fine-grained cortical parcellation.

With these outstanding results came the realization that the physical embeddedness of those networks was crucial to understand their properties. In 2013, Ercsey-Ravasz et al. (2013) uncovered a relationship between connection weights (i.e. fraction of labeled neurons, or FLN, the number of labeled neurons in an area divided by the total number of neurons labeled by the injection across cortex) and distances (i.e. the physical distance through white matter, in millimeters). This relation extends beyond mere correlation, it expresses a probability of a connection as a function of its *wiring cost*. Furthermore, this distribution follows an exponential decay, meaning that the probability and weight of a given connection between a pair of areas decreases with distance, and does so exponentially. This exponential distance rule (EDR), is reminiscent of a similar distance rule at the local scale, observed within 2 mm of retrograde TT injection sites (Markov et al. 2011).

As all exponential decays, the EDR has a unique decay parameter λ, which can be used to constrain the construction of otherwise random networks. When applied this way, the EDR is able to predict and retrieve many statistical features of the actual inter-areal network, including the proportions of absent, unidirectional and bidirectional connections (i.e. 2-node of dyadic motifs; see Box 1), the distribution of triadic (i.e. 3-node) motifs, and the distribution of cliques (i.e. complete sub-networks) as a function of their size (Ercsey-Ravasz et al. 2013). Further, the EDR has been applied in a generative random model of connectivity at the axonal level and successfully retrieves several aspects of the macaque’s actual data topology and binary properties at the inter-areal level (Song et al. 2014). As the EDR is a statistical property of the cortex, it cannot predict the existence or absence of a given specific connection. Nonetheless, it is a crucial feature that defines the topology of the cortical network and should be seen as an alternative category of networks to describe the cortex (Markov et al. 2013a).

**Fig. 1:**
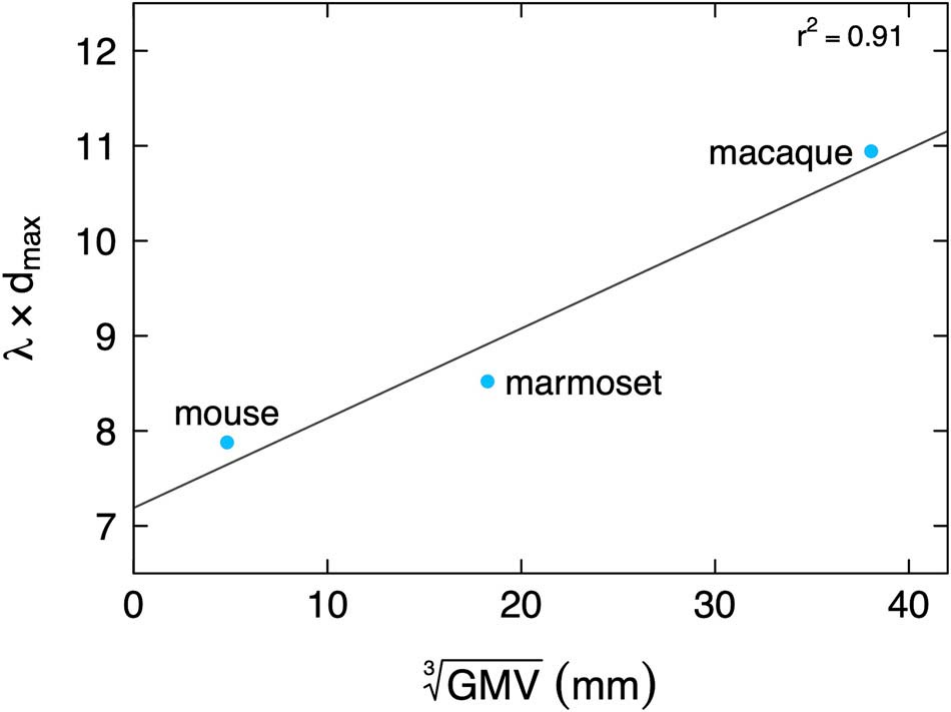
Normalized λ as a function of the cubic root of gray matter volume. The EDR exponential decay parameter λ, usually expressed in mm^−1^, is here multiplied by the maximal inter-areal distance (mm) of the atlas, rendering it dimensionless, and therefore comparable across species. This dimensionless λ increases with brain size, showing that the exponential decay of the EDR becomes sharper as brain size increases.

Subsequently, this EDR was found to exist in the mouse meso-connectome (Horvát et al. 2016; Gămănuţ et al. 2018), in the rat (Noori et al. 2017), and in the marmoset (Theodoni et al. 2021), and held the same explanatory power of network statistics in all these species (for a discussion about the human meso-connectome, see Box 2). Furthermore, it appears that the λ of each species studied thus far scales up as normalized gray matter volume goes up (Fig. 1), while density undergoes a 30% decrease from mouse to macaque (Markov et al. 2014a; Gămănuţ et al. 2018). In other words, the bigger the brain, the sharper the decay with distance relative to brain size, resulting in an increased overall binary disconnection. This aligns with Ringo’s (1991) theory that increasing neuron count while maintaining density is constrained by physical space. As neuron count grows, required connections increase exponentially, with white matter volume quickly outrunning gray matter. This is however not the case, as one of the clearest findings in comparative neuroanatomy is that the white matter volume is strictly constrained to a power law (Zhang and Sejnowski 2000). The problem is even greater in primates, where average neuron soma size does not scale up with brain volume, as opposed to rodents (Herculano-Houzel 2012; Herculano-Houzel et al. 2014; Herculano-Houzel et al. 2015). Consequently, evolving an EDR at the meso-scale becomes an almost necessary effect of natural selection. Short-range connections, energy-efficient and space-saving, face less selective pressure than long-range ones and should be more abundant, a trend that intensifies with axonal lengths.

**Box 2.** The case of the human meso-connectome.

Although neuroscience does not, in principle, revolve around the human primate, it is only fair that we should discuss it when thinking about scaling properties of the brain across species. In Theodoni et al. (Theodoni et al. 2021), the λ value for the human is extrapolated to be 0.1, based on a power law correlation between λ and grey matter volume that includes mouse, rat, marmoset and macaque. For obvious ethical reasons, the investigation of the human inter-areal connectome is for now confined to diffusion-based MRI coupled with tractography (TG) which produces non-directed, symmetrical connectivity matrices that fairly systematically tend to be complete (i.e. all-to-all connectivity) or at least very dense. We know that to be an incorrect estimate of the meso-connectome because, in comparison, Tract–Tracing (TT) produces a directed, asymmetrical matrix with sharp discrimination for absent connections.

In macaques, where both techniques can be compared, the correlation between TT and TG is 0.59 after symmetrization of the TT matrix (Donahue et al. 2016), meaning that already there is need to create false positives to increase the correlation. Further, the same study shows that the correlation drops by half when removing the 25% highest TT connections weights, which is to say that most of the correlation comes from the stronger and more easily detectable connections. At best, therefore, we know that 40% of the TG data does not really track existing connections as otherwise detected by TT, and more so for the medium/low weight connections, which are the ones that give specificity to the network. On top of that, TG is, as we said, very weak at detecting absent connections (i.e. finding true negatives vs. False positives). Unfortunately, there is no reason to think that those issues are less important in the human brain (but they could well be worse, given the size), so one should be very careful about conclusions that can possibly be drawn from human dMRI-based TG, at least regarding the network’ shape, statistical properties and topology.

That cautionary tale now properly laid out, a recent study from Rosen & Halgren (Rosen and Halgren 2021) extracted a TG-based λ value for the human brain based on the collated and massive dataset of the Human Brain Project. Defying the prediction from Theodoni et al, the authors find a human λ value of 0.04. meaning that if they are correct, the human meso-connectome EDR would be even more abrupt than what is predicted by extrapolation from other species. The potential effect of such a low value in a big brain (human or not) is laid out and discussed in the section called "Evolution of cortex topology" of the main text.

Before closing this boxed section, we ought to mention another promising line of investigation (Yeo et al. 2011) is using what is known as functional connectivity MRI (fcMRi), based on a detailed analysis of the Default Mode Networks (DMNs), where synchronized activations show potential to be a good predictor of TT connectivity, and vice-versa (Buckner and DiNicola 2019). As is the case for the TG, nonetheless, connections characterized as weak by TT may elude detection as they would fail to ignite activity in the corresponding area.

Additionally, the EDR not only optimizes brain wiring but also enhances *evolvability,* in that it facilitates the next evolutionary step. In EDR-based cortical networks, adding long-distance connections incurs minimal cost and doesn’t necessitate extensive rewiring, paving the way for future cortical expansion (Markov et al. 2013a). This underscores the importance of λ as a critical evolutionary factor fine-tuned by natural selection. To be exact, evolution selects for cost-effective, self-organizing information processing system that minimizes wiring expenses. EDR-based models, with their λ parameter, effectively capture the statistical properties of this wire minimization.

### Modularity and the reach of primary sensory areas

**Fig. 2:**
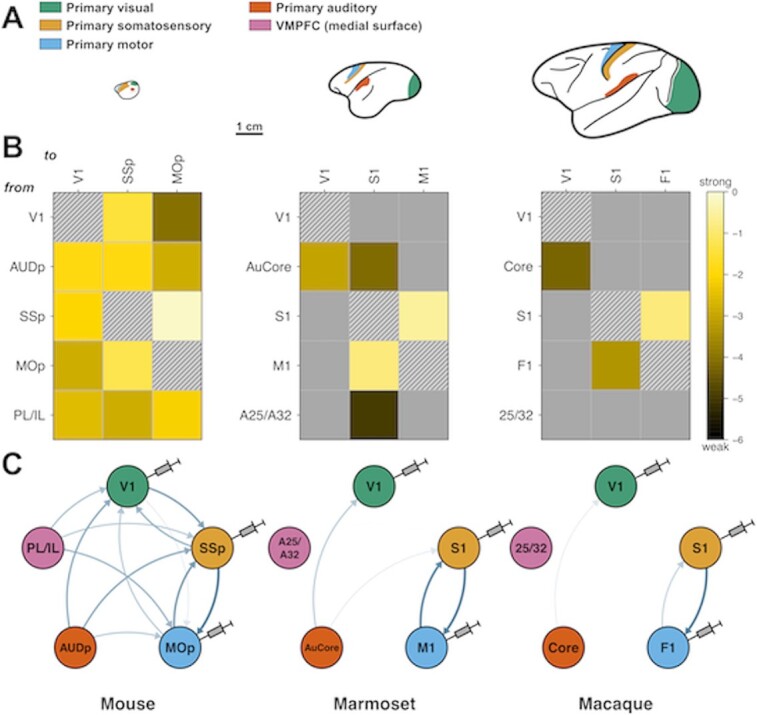
Primary sensory and motor areas become more disconnected from other primary cortical areas as brain size increases across species. (A) Lateral view drawings of mouse, marmoset and macaque brains, shown at the same scale. Colors indicate analogous primary areas across the three species for which connectivity data are available: green, visual; orange, somatosensory; blue, motor; red, auditory; and pink, ventromedial prefrontal cortex (not shown). Bar scale, 1 cm. (B) Reduced retrograde tract-tracing, inter-areal connectivity matrices for each species, resulting from injections in primary visual, somatosensory, and motor (columns), and showing 5 source areas (rows): V1, primary visual; AUDp/AUCore/Core, primary auditory; SSP/S1, primary somatosensory; mop/M1/F1, primary motor, PL/IL prelimbic/infralimbic (mouse) and its primate equivalent area 25/32. Each colored entry represents a connection from a row area to a column one. Connection weights are expressed in log10 scale, ranging 6 orders of magnitude, from dark brown (very weak) to bright yellow (very strong). Gray represents absent connections and hatched squares, self-connections. (C) Circular graph representation of connectivity data shown in (B), with color-coding from (A). Injected areas are depicted with needles. Arrow color intensity and width indicate weights: thin pale arrows: weak connections; thick dark blue arrows: strong connections.

A striking example of brain scaling properties is the distance, relative to brain size, reached by a given area's connectivity. In the mouse, as we have seen, a 0.97 density means that, with few exceptions, all cortical areas are connected with one another. This is not to say that there is no wiring cost pressure, even at this brain size. The very fact that the mouse has an EDR precisely means that there is already a higher cost to long connections. Still, an area such as V1 receives and sends inputs to all areas so far injected, including other primary cortical areas, as well as motor, premotor and limbic cortices (Gămănuţ et al. 2018; Fig. 2). Mouse V1 is, in terms of connectivity, far from being a purely visual area, could be considered to be associative, as with every other mouse cortical area.

In comparison, primate V1 has very restricted connectivity. Marmoset or macaque alike, V1 connects to only about 45% of all areas. The prefrontal cortex, mostly nonexistent in the mouse, is largely disconnected from primate V1, along with motor and premotor cortices. In fact, primate V1 primarily focuses on extensive connections within the visual system (Markov et al. 2014a; Theodoni et al. 2021). V1 is in these datasets only represented by V1 foveal injections, but still retains in the macaque weak connections with the auditory cortices (Markov et al. 2011), such as medial and lateral belt (MB and LB, equivalent to a secondary auditory cortex), the parabelt (PB; tertiary auditory) and even a few detected cells in the Core (primary auditory cortex). Injections in the far eccentricities of V1 reveal important connections with the core, belt and parabelt areas, opening further the possibility of mild multimodal integration in primary sensory areas (Falchier et al. 2002).

Not without caution (larger relative size and barrel field), a similar pattern emerges for S1 (primary somatosensory cortex, Fig. 2), highly interconnected with nonsomatosensory/nonmotor cortices in the mouse, while only mainly connected to the motor, cingulate and insular cortices in the primate. There are also some connections to higher level dorsal stream areas, consistent with the *vision of action* paradigm, as described by Goodale and Milner (Goodale and Milner 1992; Goodale 2011).

These comparisons are only tentative, as many anatomical, physiological and ecological differences separate mice from primates (e.g. nocturnal/diurnal lifestyles, eye orientation, binocular vision, no fovea nor visuotopy in mouse V1; Ohki et al. 2005; Ibbotson and Jung 2020). Furthermore, rodents and primates have elected for vastly different evolutionary strategies, shaped by and in shaping radically different ecological niches, such as highly socially complex arboreal life vs socially simple burrowing ones, for which visual systems are bound to be adapted (Previc 1990).

Nonetheless, within reason, the analogy between primary visual and somatosensory cortices of rodents and primates stands, and they sharply exemplify what happens to all areas when transitioning from rodents to primates. The “connectivity horizon” reduces (in a normalized brain perspective), from the entire cortex in the mouse, to a more specific pattern reflecting the involved functions. In graph theoretical terminology, the cortical network becomes more modular (see *modularity*, Box 1), meaning that the community structure, if always present in weighted terms, becomes clearly delineated even in binary ones. This, we argue, is an essential aspect of applying an EDR to a larger brain. As the cortex expands, wiring costs increase differentially on short-range and long-range connections. Thus, network density falls as long-range connections are selected out, leading to a specific and modular pattern of connectivity.

### Evolution of cortex topology

Based on what was just said, one could conjecture that, applied to very large brains such as apes and the human primate, density would continue to fall and modularity to increase, up to a point where the cortex would essentially be a sparse network of function-oriented communities, largely isolated from one another, apart from the few connections linking them together. In short, a small-world network, with its collection of hubs. That would be, however, a bad approximation of the EDR applied to large brains.

**Fig. 3:**
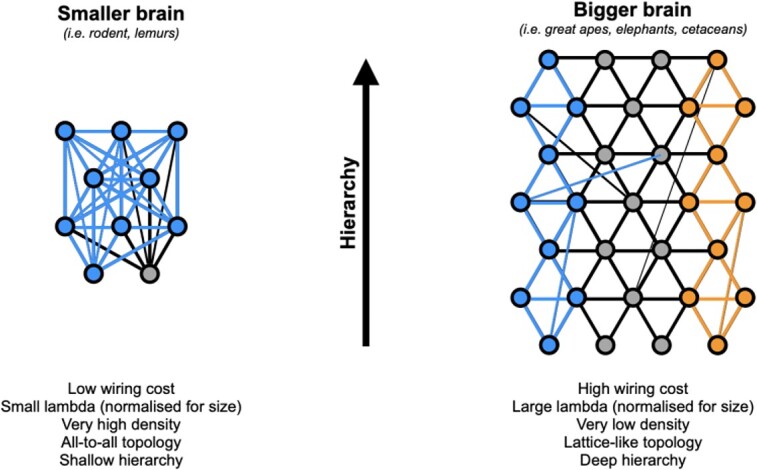
Effect of brain size on the topology of the inter-areal network. In smaller brains with lower wiring cost, an EDR-based brain with a smaller (normalized) λ parameter leads to an “all-to-all,” (near-) complete graph (i.e. very high density), as shown by retrograde tract tracing data. In such brains, information propagates from sensory primary areas to the entire network very quickly (blue connections), with little room available for hierarchical information processing or segregation of function. In bigger brains such as great apes, cetaceans, or elephants, the increased wiring cost leads to a larger (normalized) λ and a correspondingly sharper EDR. Density falls drastically (here chosen at 0.07 for illustration), leaving place to a much sparser network. We posit that, in such sparse network, we should expect a *k*-NN topology akin to a lattice, where each area is primarily connected to its direct neighbors and only a few longer-range connections otherwise remaining. There, information would spread gradually, from neighbor to neighbor, allowing both hierarchical processing and segregation of function (blue and orange connections). Intermediate size primate brains, such as marmosets or macaques, would fall in-between those two ends of the spectrum.

However big a brain, and however large a λ value, each area will always be connected to their most direct neighbors. So we propose that the global topology one gets when increasing size of an EDR-constrained brain size is not one of a small-world, but one of a *k*-nearest neighbor network (*k*-NN, which we will here loosely call a lattice-like network, as such a network would gradually converge to a lattice as the number of neighbors, *k,* diminishes), where any area is always connected to its local physical neighbors, with rarefying middle range-connections, and even rarer long-range ones (see Fig. 3). This is highly consistent with the calculations of Murre and Sturdy (1995) where a nearest neighbor topology gives very small connectivity (i.e. white matter) volume. In fact, it could easily be argued that small brains such as the mouse are also of k-NN topology, only with an arbitrarily large *k.* In that kind of topology, the cortex becomes akin to a sheet of gradually changing functional properties and functional segregation becomes increasingly possible as the brain increases in size and the lattice structure takes over from the edge-complete structure of smaller brains, thus offering a path to parallel processing, such as the dual visual stream system.

Although the notion of a dual visual stream system is well documented in primates (Mishkin et al. 1983; Goodale and Milner 1992; Kravitz et al. 2011) and appears to be reaching consensus for the mouse cortex (Harris et al. 2019; D'Souza et al. 2022), details such as which functional aspects are segregated are still very much under investigation (Wang et al. 2012; Glickfeld and Olsen 2017; Saleem 2020; Gămănuţ and Shimaoka 2022). There is also a weaker hierarchical organization in mouse than in primates, consistent with the more dense connectivity at earliest stages in mouse (Gămănuţ and Shimaoka 2022). Assuming we are correct, an EDR-based, small-brain topology, close to edge-completeness, could still harbor some aspects of dual processing, based on poor but existent segregation (i.e. weak but non-null community structure), with porous functional boundaries, as it indeed appears to be the case (Gămănuţ and Shimaoka 2022).

Another example of segregation of function and increased hierarchical complexity with respect to primate brain size can be found in the somatosensory/motor systems. The two systems stay well connected together across evolution (Figure 2) and, in fact, stay also fairly close together in terms of their spatial proximity on the cortical mantle. This should not be surprising in the context of an EDR-based network: if two areas close together will probabilistically share a strong connectivity, the converse could also well be true, such that two areas that require tight connectivity to perform their functions will stay close together through evolution. Indeed, the somatosensory and motor systems, although described with different names, are actually operationally interdependent, with the somatosensory system providing proprioceptive information that is essential to motor function. Thus, this may be an example of areas being adjacent to one another due to their inseparable functions. Further, the EDR would predict that the bigger the brain, the more stuck together they are. As the normalized λ increases with brain size, the "connectivity horizon" shrinks in normalized space, forcing together any two areas that require high connectivity. In actual space, however, it remains true that a bigger brain means more space for a given system to produce new and/or more specialized areas whose network, thanks to a very sharp EDR, will soon exhibit highly specific connectivity. Jon Kaas, in his excellent 2004 paper (Kaas 2004) on the evolution of the motor system in primate, reported increasing modularity and complexity in connectivity patterns within the somato-motor system, and already suggested there that modularity would be a valid solution to the wiring problem in large brains.

Of course, size cannot possibly be all there is regarding brain evolution. Cortex expansion has happened independently numerous times during mammalian evolution, leading to different cortical organizations (Northcutt and Kaas 1995), with, at the heart of it, differential growth and timing in different cortical territories (Finlay 2009; Finlay and Huang 2020), resulting in a conserved pattern of cortical expansion across species (Chaplin et al. 2020). The capybara, largest rodent on earth, has a brain of about 60 g, barely less than the cynomolgus monkeys from which the TT data are produced. Its gyrified cortex, indicative of increased skull constraints, is, as for all rodents, almost completely devoted to sensory and motor fields rather than association cortices (Campos and Welker 1976). Nevertheless, an EDR should remain at work, allowing potentially for a similarly dense connectome to that of the cynomolgus and a similar topology. Conversely, the mouse lemur, the smallest primate known to exist, has a lissencephalic brain of 1.8 g, similar to that of a rat. There, an EDR would likely predict a highly dense inter-areal connectivity like that of the mouse, but with a functional layout of a primate. Segregation of function and community structure, as with the mouse, should be weak but not necessarily completely absent. Recent functional imaging data in the mouse lemur are compatible with this interpretation (Garin et al. 2021). In short, size will determine the topology, and thus network capacity for functional segregation, but not the specific function that emerges from a given cortical area.

When applying evolutionary logic to the brain, it is often assumed that the increase in size *led* to higher and more complex cognitive functions, that it opened a new ecological niche, to *only then* adapt brain functions to it. Although very much valid, we would like to complete the picture by offering here that the primate brain can also and nonconcurrently be explained by reversing this evolutionary causality. In particular, we submit that cortical expansion happened *because* complex cognitive functions were needed to survive and reproduce. Perhaps because primates entered a niche of heightened sociology and arboreal life (Ho et al. 2021), they needed at the same time an efficient understanding of spatiality (Previc 1990) and social/political interactions (MacKinnon and Fuentes 2011; Gintis et al. 2019), both of which require extensive memory skills (Where is the food? Who has it? Are they friends or foes? etc.; Sherry and Schacter 1987; Klein et al. 2002; Allen and Fortin 2013), abstraction abilities (inter-individual interactions as objects, hierarchy, and political rule inference; Klein et al. 2002), planning and compositionality (Byrne 1998), as well as some amount of theory of mind, or at least its early precursor (Brüne and Brüne-Cohrs 2006). Selecting for brains capable of all this seems to mean, at least in part, the implementation of function segregation and parallel processing. Evolution tends to follow the easier route, and a bigger brain, already constrained by an EDR since at least the last common ancestor with rodents, ontologically gives all that, as we have seen. Although more energy-consuming, a larger cortex should be evolutionarily easier than a careful rewiring of a small brain because the number of necessary mutations is minimal (e.g. vary one parameter governing neuronal proliferation, rather than change all the signaling events that guide connections to their more permanent configurations). This is all the more easy, in fact, since the EDR also grants the cortex a fair amount of *evolvability*, as it makes the next evolutionary step easier to achieve (Markov et al. 2013a). It stands to reason, therefore, that one solution evolution would employ to address the need for complex cognitive skills is precisely a brute force increase in brain size. This is certainly highly consistent with the “social brain hypothesis” (Dunbar and Shultz 2007), which argues that social group size, and its associated cognitive demands, is one of the prime factors driving brain expansion.

## Cortical hierarchies across species

Increasing modularity in network topology is not the only salient distinction between mice and primates that may allow for the emergence of abstraction and higher cognition. In addition to a smaller brain, mice have a shallower hierarchical organization than primates (Goulas et al. 2017; D'Souza et al. 2022; Gămănuţ and Shimaoka 2022) and a narrower range of cortical heterogeneity (Charvet et al. 2015; Fulcher et al. 2019; García-Cabezas et al. 2023)*.* Additionally, scaling factors do not apply equally to all cortical areas. Association areas have disproportionately expanded in the primate lineage (Finlay 2009; Chaplin et al. 2020). Below, we will elaborate on how hierarchies are defined and discuss species differences.

### What is a cortical hierarchy?

Cortical hierarchy depends on the concept of directionality and sequence in connectional architecture. In early studies of the macaque visual system, physiological and anatomical evidence suggested that projections with “top–down” functional correlates were characterized by inter-areal anatomical connections with the majority of origin neurons located in deep layers V to VI, which were deemed “feedback.” We will elaborate more on top–down functional correlates in a later section. The converse “bottom–up” functional correlates were characterized by inter-areal anatomical connections with the majority of origin neurons located in superficial layers II to III, deemed “feedforward” (Rockland and Pandya 1979; Rockland 2022). Further study elucidated that not only did the origin neurons in such pathways have distinct laminar assignments, but their terminations also populated distinct laminar compartments. For example, feedback pathways typically featured a wide axonal termination zone in layer I with a notable absence in layer IV, and feedforward pathways had a distinctive termination zone in layer IV, like canonical sensory thalamo-cortical projections (reviewed in: Vezoli et al. 2021; Rockland 2022). Thus, “top–down” and “bottom–up” functional processes could be correlated with distinct anatomical relationships. Later studies demonstrated that cortical connectivity is frequently reciprocal, with a feedforward projection returned by a feedback connection.

But rather than being interchangeable terms, “top–down” and “feedback,” or “bottom-up” and “feedforward,” are best understood as dissociated concepts from distinct modalities (function and structure, respectively), but which can be useful to pair together conceptually. One reason for this distinction is that inter-areal processes considered to exemplify top–down roles are not uniform in feedback anatomical presentation. Inter-areal anatomical connections rarely feature connections originating only in superficial (II to III) or deep (V to VI) layers, in fact, they are often graded. For example, consider two canonical feedback projections: (i) from V2 to V1, where an average of only 58% of neurons are found in deep layers, while 42% of neurons originate in superficial layers, and (ii) from V4 to V1, where ~70% of neurons originate in the deep layers (Markov et al. 2014b; Chaudhuri et al. 2015). Another reason is that feedback and feedforward connections also exist outside the sensory systems. For example, a recent study suggested that the macaque subgenual prefrontal area 25 has a strongly feedback relationship to prefrontal area 12 (Joyce and Barbas 2018), a feedback presentation that does not fit neatly into a “top–down” functional framework as conceived in sensory systems.

Hierarchy can be defined in several distinct ways (Hilgetag and Goulas 2020). One definition follows the example set by Felleman and Van Essen (1991), where feedforward and feedback relationships are used to assemble a sequenced structure of information flow with feedback-dominant structures at the top and feedforward-dominant structures on the bottom, though this hierarchy had critical drawbacks in its initial conception (Hilgetag et al. 1996). This concept of hierarchy has since been further constrained by the integration of connectional weight decay over distance to produce a refined version, via the Kennedy/Knoblauch group (reviewed in Vezoli et al. 2021). These efforts have produced a sequenced network superstructure assembled from empirically obtained weighted feedforward and feedback indices obtained using a uniform pipeline of retrograde injections and analyses in 40 areas of the macaque (and studied in 91 areas; Froudist-Walsh et al. 2021), an enormous feat, though as yet incomplete given the exhaustive effort required to add cortical areas to it. We will refer to this as the anatomical hierarchy. This pipeline has been extended to the marmoset (Theodoni et al. 2021) and mouse (D'Souza et al. 2022; Gămănuţ and Shimaoka 2022) and, as we have seen for network topology, now allows a direct species comparison of anatomical hierarchy as well. In such comparisons, marmosets exhibit a similarly deep hierarchical structure as the macaque, with some notable exceptions, while mice exhibit a shallower hierarchy.

A shallow hierarchy can be distinguished from a deep hierarchy in a few ways. To understand this distinction, it is necessary to introduce the concept of feed-lateral or columnar connections. This type of connection falls in between a feedforward or feedback presentation, where close to equivalent proportions of neurons (at origin) are located in supra- and infra-granular layer compartments. The return connection is typically also feed-lateral, with terminations appearing across layers (Felleman and Van Essen 1991). Cortices with reciprocal feed-lateral connections are positioned within the same hierarchical level, while cortices with a reciprocal feedforward-feedback pattern are positioned in distinct hierarchical levels. A shallow hierarchy occurs from the detection of minimal hierarchical steps within the network, while a deep hierarchy occurs from the detection of many steps within the network.

A distinct concept, but one which is sometimes paired with anatomical hierarchy, is that of a cortical gradient or spectrum, which can be any changing parameter along the cortical surface, for example, cytoarchitectonics (John et al. 2022), or functional connectivity (Margulies et al. 2016). Recent lines of evidence suggest that some gradients can align with the current sequence of the anatomical hierarchy in primates, such as receptor density (Froudist-Walsh et al. 2021; Froudist-Walsh et al. 2023; Zilles and Palomero-Gallagher 2017; Goulas et al. 2021; Hansen et al. 2022), intrinsic timescale and mRNA expression (Murray et al. 2014; Burt et al. 2018), or spine density (Elston et al. 2011; González-Burgos et al. 2019; Theodoni et al. 2021), implying the possibility that there may be an alignment of laminar patterns of connections with larger forces that determine cortical gradients in primates, and to a lesser degree, in mice as well (Fulcher et al. 2019; Gămănuţ and Shimaoka 2022). A framework uniting gradients in cortical features and the laminar pattern of connections called the structural model (Barbas and Rempel-Clower 1997) was based on the observation in 1986 by Helen Barbas (Barbas 1986) that the degree of difference in cortical type between connected areas can predict the presence and strength of laminar pattern of connections (Beul et al. 2017) and sometimes is now also called the architectonic type principle (Hilgetag et al. 2019).

### Linking cortical type, laminar connections, and species differences

The structural model can also be used as a theoretical framework for cross-species comparisons (García-Cabezas et al. 2019; Goulas et al. 2019). Architectonic type is often presented as an ordinal variable akin to dimensionality reduction of multiple lines of neuranatomic evidence, including myelination, laminar elaboration, and other chemoarchitectonic factors (Barbas and Rempel-Clower 1997; Dombrowski et al. 2001; John et al. 2022), though type can often be approximated by neuron density if a continuous variable is desired (Medalla and Barbas 2006; Beul and Hilgetag 2019). The structural model demonstrates that if two connected cortical areas are of similar architectonic type, they exhibit a feed-lateral or columnar pattern of reciprocal connections, meaning participation of superficial and deep layers at both the sender and receiver cortex. Because the cortex is a gradually changing structure, areas of similar cortical type are often nearby in terms of wiring distance, and these as we have seen are the strongest connections by traditional neuranatomic measures (e.g. FLN). Two connected cortical areas have more polarized laminar patterns of connections when they are more dissimilar in cortical type, meaning that two very dissimilar cortices exhibit a more pure feedforward and reciprocal feedback architecture (e.g. even small changes in cortical architecture are correlated with subtle shifts in laminar connectional architecture (Medalla and Barbas 2006). The link between laminar connectional architecture and relational cortical architecture is hypothesized to emerge from developmental forces (Barbas 2015; Beul and Hilgetag 2020) that may together shape cortical architecture, laminar patterns of connections, and possibly the wiring length of connections (García-Cabezas and Zikopoulos 2019).

The phylogenetic expansion of primate cortex includes an expansion in the range of cortical type, such that the most elaborate primary sensory areas in macaques (e.g. V1) do not resemble in laminar sophistication their counterparts in rodents, while the least elaborated cortices (e.g. limbic cortices) have more recognizable counterparts in rodents (García-Cabezas et al. 2023). According to the structural model, with increasing cortical heterogeneity comes increasing combinatorial possibility for laminar patterns of reciprocal connections, and thus increasing opportunities for transformation of signals in segregated streams of a modular network. Given the narrower range of cortical heterogeneity in mice, the structural model predicts more feed-lateral type connections and fewer pure feedback and feedforward relationships, i.e. a shallower hierarchy, which appears to be true (Harris et al. 2019; D'Souza et al. 2022; Gămănuţ and Shimaoka 2022), although there are some critical distinctions, for which more widespread adoption of single-neuron tracing resolution technology in the macaque could help address (e.g. Zeisler et al. 2023). Thus, the larger diversity in cortical types and laminar connections in macaque may confer a deeper hierarchy and more opportunities for refinement of information.

Macaque and mouse V1 differ starkly in laminar organization (Gilman et al. 2017; García-Cabezas et al. 2023), perhaps owing to the expanded proliferation during development that is well documented in primates (Dehay et al. 1993; Kornack and Rakic 1998; Smart et al. 2002), leading some to argue that they are not homologous structures (García-Cabezas et al. 2023). Like cytoarchitectonic type, which is generally assessed using laminar elaboration and some chemoarchitectonic parameters, microscale structural and physiological data also exhibit a wider range of heterogeneity across the cortical landscape in macaques than in mice, indicating that not only is there more modular “space” in network topology for specialized processes but also more specialization in the structural and physiological attributes of the cortices that carry out these processes.

A few studies in the last decade have demonstrated this point. Comparative studies have revealed that layer III pyramidal cells in frontal cortex and V1 of mice have roughly equivalent morphological and physiological properties, while in macaques, there are stark differences in these properties between prefrontal cortex and V1 (Amatrudo et al. 2012; Medalla and Luebke 2015; Gilman et al. 2017). Neurons in mouse V1 and frontal cortex have higher synapse density, more total spines, higher spine density, and larger and more complex dendritic arbors than neurons in macaque V1, but are lower than macaque dorsolateral prefrontal cortex (dlPFC) neurons (Gilman et al. 2017; Wildenberg et al. 2021). Layer III pyramidal cells in primates in general exhibit greater morphological heterogeneity across the cortical expanse, with the highest reported dendritic complexity and spine density found in deep layer III of dlPFC and anterior cingulate, compared to V1 and early visual cortical areas (Elston 2000, 2003; Elston et al. 2006; Elston et al. 2011; Medalla et al. 2017), while mice have no detectable gradient in spine density variation among areas so far studied (Ballesteros-Yáñez et al. 2006; Gilman et al. 2017). The gradient of increasing spine density along the ascending anatomical hierarchy in primates is central to recent theories of primate working memory, which have been successful at replicating the partially distributed patterns of persistent activity seen during working memory tasks in modular networks (Wang 2020; Froudist-Walsh et al. 2021; Mejías and Wang 2022). Further, comparative ultrastructural analyses have revealed that macaque dlPFC axospinous synapses are characterized by larger presynaptic boutons and postsynaptic densities including a greater proportion of perforated synapses, compared to those in V1 (Hsu et al. 2017). Thus, there are more, and presumably stronger, synapses on layer III pyramidal cells in dlPFC than V1, while there are relatively more short, “stubby” spines in macaque V1 than in dlPFC (Amatrudo et al. 2012; Young et al. 2014). As shorter spine necks are associated with more accurate and rapid transfer of synaptic excitation to the parent dendrite (Araya et al. 2006), the increased number of stubby spines in macaque V1 would be consistent with more faithful neurotransmission that may be a critical in early sensory cortices, but more detrimental to more complex computations required to maintain persistent firing for abstraction and working memory. Together, this suggests that there may be less diversity in laminar elaboration and pyramidal cell morphology in mouse cortex compared to primate cortex. In particular, mouse cortical areas studied thus far seem to lie between the extremes of the macaque cortical diversity gradient, with neither signature of primate primary sensory cortex nor higher hierarchical regions like dlPFC.

In summary, primates have more modular cortical networks, allowing for distributed communities that can maintain degrees of relative information segregation, which may allow information to be selectively combined and integrated. Cortical heterogeneity may allow further specialization to occur. Separately, a deep hierarchy allows iterative feedforward and feedback interactions, thought to provide the basis for belief update in the framework of active inference (Gregory 1997; Friston 2010). In contrast in mice, a dense network topology produces a comingling of information at all (though fewer) levels, and fewer cortical areas, with less structural diversity, mean fewer opportunities for specialization and inter-areal refinement. In the following sections, we will discuss the consequences of species differences in meso-connectome topology by reviewing V1 physiology and then the interaction between the cortex and the hippocampus. We will then elaborate on primate prefrontal specializations for working memory and the evolution of molecular mechanisms.

## Consequences of species differences in multisensory integration and network topology

### Multisensory convergence in primary areas across network topologies

A dense connectome, such as observed in the mouse, means that almost all areas are connected to each other, including early sensory areas. This specific configuration seems central to facilitate early multisensory integration, in which congruent multimodal stimuli are known to decrease reaction time compared to unimodal stimulus (Molholm et al. 2002; Sakata et al. 2004; Wang et al. 2008). This may be especially helpful to the survival of small animals—with necessarily small brains—that are frequently preyed upon, where their rapid response to threatening events would be paramount. Early convergence of sensory information might facilitate initiation of fast, “instinctive” motor responses necessary to navigate threatening situations (freezing, fight, flight, etc.).

Additionally, the formation of a long-term memory of the emotional event could help to guide more effective, future decisions, such as when to avoid vs. approach. For example, bringing together both the sound and sight of a hawk in area V1, coupled with simultaneous somatosensory information regarding whether one is exposed vs. hidden, could provide an early signal about how to respond to a dangerous event. Similarly, the formation of long-term memories regarding dangerous vs. safe contexts has clear survival benefit, where multiple sensory aspects form a detailed memory. In humans, this is described as episodic memory, where the convergence of sensory, affective, and cognitive events over an interval of time conjoin to create a fully integrated “episode,” which is then consolidated into longer-term memory if it is sufficiently salient or meaningful. This type of memory relies on the hippocampal formation, which we will elaborate upon in a later section (Scoville and Milner 1957).

Of course, primates also rapidly respond to threatening situations. Larger brains, such as the primate brain, feature networks that are more modular, with connections between early sensory areas largely absent. This endows the network with the flexibility to integrate sensory information later on, but it does not mean multimodal information can never reach primate primary sensory areas, because this can occur via feedback projections from higher areas back to early areas. The modularity in a primate cortical network instead may confer (i) the ability for sensory information to be transformed and refined before large-scale multimodal integration, and (ii) the flexibility to use more refined multimodal sensory information to inform early sensory processes, if needed. For example, there is evidence in humans and monkeys that multimodal information can affect processes in primary sensory areas (Driver and Noesselt 2008). In addition to feedback cortical connections, multimodal information that reaches unimodal areas may also be conveyed via thalamic connections (e.g. the pulvinar nucleus, Froesel et al. 2021). There is also some retained early modality integration in pathways where rapid synthesis is essential, for example, when early visual responses are modulated by eye movements to prevent blurred vision (Denagamage et al. 2023) and for coordination of head and eye movements with vestibular and visual information (Angelaki et al. 2011). Finally, neuromodulation, the subject of our last section, can rapidly silence the activity in primate lateral prefrontal areas that are necessary for abstraction. This is thought to be a mechanism to divert resources to processes more relevant to multimodal integration and rapid defensive behaviors (Arnsten 2009; Arnsten 2015).

### Cortical topology and a comparison of V1 *in vivo* neuronal physiology in the macaque vs. the mouse

Consequences of varying cortical network topology across mice and macaques can be seen in studies using *in vivo* electrophysiology. Decades of neurophysiological recordings from area V1 in primates have shown that these neurons respond selectively to visual features (e.g. color, orientation, motion direction). Area V1 neurons show selectivity for stimuli presented on the retina even in anesthetized animals (e.g. Hubel and Wiesel 1968), while V1 responses to other sensory modalities have been reported in awake macaques for certain tasks that require multisensory integration (Wang et al. 2008), but these are restricted to the peripheral visual field that receives some auditory projections (Fig. 2 and Falchier et al. 2002).

Although the macaque and mouse recording paradigms have many differences, they usually involve head fixation for stable recording, but the ability to move the body in a chair (primate) or spherical treadmill (mouse). Given this similarity across paradigms, and the large species differences in V1 cortical connectivity described above, it is particularly interesting that macaque V1 neurons show little or no response to the monkeys’ movements (Talluri et al. 2022), while a large number of studies show that mouse V1 neurons are greatly influenced by locomotor state (Ayaz et al. 2013; Saleem et al. 2013; Erisken et al. 2014; Dadarlat and Stryker 2017; Dipoppa et al. 2018; McBride et al. 2019), such as enhancing the encoding of visual stimuli (Dadarlat and Stryker 2017). While additional studies in macaques with larger-scale movements would be needed for a direct comparison to the mouse paradigm, the physiological data so far are consistent with the lack of projections from somatosensory and motor cortices to V1 in primates rendering V1 neurons selectively responsive to visual stimuli and the presence of projections from S1 and M1 to V1 in mice providing large effects of locomotion on visual responses (Fig. 2).

There are multiple caveats that must be considered when comparing visual physiological responses across species. An obvious species difference is that primates are diurnal and highly visual, while rodents are nocturnal and rely more on olfaction and somatosensation. As mouse vision is adapted to a nocturnal ecology, they do not have a fovea or perceive color. Their eyes are located on the sides of the head to maximize the visual field, but thus they have poor stereo vision. Indeed, mice can move their eyes independently (Michaiel et al. 2020). Although these adaptations are consistent with a nocturnal vs. diurnal animals, they cannot account for the differences in cortical connectivity between rodents and primates described above, as species differences in cortical connectivity are not unique to V1 (they are evident in the pattern of connections of S1 as well, Fig. 2). There are also important differences in the earlier aspects of the visual system between mouse and macaque that should be considered, for instance the more extensive processing of visual stimuli by the mouse retina than by the primate retina, and the differences in inputs to V1 (predominately from LGN in primate vs. superior colliculus in mouse). However, the example of locomotion influencing V1 activity in mice but not macaques seems to capture the differences in cortical connectivity rather than these other large species differences. We should also emphasize that the extraordinary experimental tools available for studies of mouse cortex may be beneficial for understanding primate peripheral vision, with which mouse vision shares many similarities (Niell and Scanziani 2021).

Studies in macaques have suggested that V1 records a “faithful” reflection of the visual signals entering the retina, encoding the physical visual stimulus, with only minor modulations of activity depending on whether the stimulus is perceived or not (Leopold and Logothetis 1996; Lamme et al. 2000; van Vugt et al. 2018). Another example of this phenomenon is the segregation of motion detection processes among the dorsal stream areas in primates, where V1 detects local motion signals, but the integration of motion components into global patterns occurs in higher-order areas (Lempel and Nielsen 2019). This is in contrast to mice, where motion integration occurs in V1 (Movshon et al. 1983; Smith et al. 2005; Palagina et al. 2017). The intrinsic physiological properties of primate V1 neurons are also consistent with more faithful neurotransmission, where spontaneous excitatory postsynaptic currents (sEPSCs) are diminished in amplitude and frequency and have faster kinetic profiles in layer III of macaque V1 than in dlPFC (Amatrudo et al. 2012; Zaitsev et al. 2012; Medalla and Luebke 2015). In this view, V1 captures the “raw data,” maintaining the trace for only a very brief time (e.g. ~200 ms) in iconic memory stores. As described below, such signals can be influenced by signals from higher-order association areas that feed back to V1 to serve object recognition or implement biases in signal processing according to behavioral goals (Roelfsema et al. 1998). However, large-scale multisensory integration is unlikely to occur in primate area V1, but in areas farther downstream.

### Significance of the hippocampal system as an ancestral multimodal structure

**Fig. 4:**
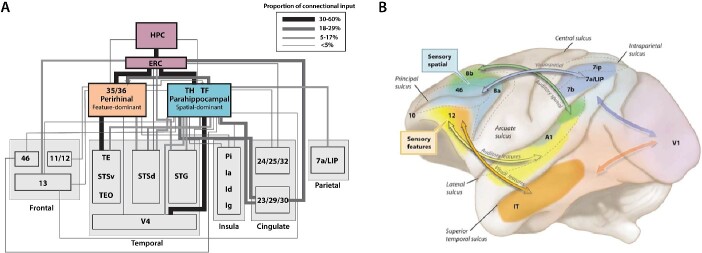
Examples of multimodal convergence and functional segregation in the macaque cortex. (A) Wiring diagram depicting inputs to perirhinal (areas 35 and 36) and parahippocampal cortices (areas TH and TF), which are major inputs to the entorhinal cortex (ERC), the main cortical entry point into the hippocampus (HPC). Inputs to the perirhinal and parahippocampal cortices are higher order association areas, and often polymodal. The perirhinal areas are more associated with feature-specific inputs and functions, while the parahippocampal areas are more associated with spatially related inputs and functions. Adapted from Suzuki and Amaral (2004). (B) The separation of sensory streams for spatial vs. feature information for both visual and auditory information continues into the PFC, terminating in dorsal (spatial) vs. ventral (feature) zones of the macaque dlPFC as conceived by Patricia Goldman-Rakic; adapted from a drawing by Mark S. Williams in (Arnsten 2003). Recent work suggests that auditory zones extend to the frontal pole and medial surface as well (Medalla and Barbas 2014).

In mammals, high-order multisensory signals converge into the entorhinal cortex, which is the entryway to the hippocampal complex (Fig. 4A) and sits atop the traditional primate visual hierarchy in the now classic wiring diagram (Felleman and Van Essen 1991) and its alternate or updated versions (Hilgetag et al. 2016; Vezoli et al. 2021). Information from cortical areas funnels through the entorhinal cortex into the hippocampus, with multisensory responses especially evident in CA3, in both rodents and primates. Current theories propose that mixed selective neurons in the hippocampus (Ravassard et al. 2013) integrate information from different sources and sensory systems such as distal visual cues (Muller and Kubie 1987), self-motion cues (Gothard et al. 1996), e.g. proprioception, optic flow, and vestibular cues (Stackman et al. 2002), and other sensory cues (Save et al. 2000), such as olfaction (Wood et al. 1999), audition (Itskov et al. 2012), and somatosensation (Young et al. 1994).

In rodents, the vast majority of hippocampal neurons in CA3 and CA1 are place cells, signaling the location of an animal in allocentric space (Moser et al. 2015). Place cells are thought to form during spatial navigation when rodents “path integrate” and hippocampal neurons integrate inputs from entorhinal cortex grid cells that respond to proprioceptive, vestibular, somatosensory, and possibly other cues (Hartley et al. 2000; Moser et al. 2015). Bonafide place cells, however, have not been found in primates (Mao et al. 2021; Piza et al. 2023). Instead, in primates (including humans), hippocampal neurons respond to a wider variety of cues (Graves et al. 2023). Nevertheless, a large proportion of cells seems to be driven by complex associations of visual inputs (Rolls and Wirth 2018; Gulli et al. 2020; Corrigan et al. 2022; Piza et al. 2023).

These species differences in selectivity of hippocampal neurons could in part be a result of cortical network topology differences. For example, the mouse hippocampus receives more “raw” sensory information than the monkey, because the mouse entorhinal cortex is directly connected with primary sensory areas. On the other hand, in primates, entorhinal connectivity with early sensory areas is weak, yet it is stronger with association areas that are themselves farther removed from primary sensory areas (Fig. 4A; Bergmann et al. 2016). Thus, the primate hippocampus may receive sensory information that is significantly more transformed than the mouse hippocampus, which may, in turn, result in a more selective dialogue between the hippocampus and neocortical areas during memory consolidation. Sensory signals that make it into in the “less” early sensory-connected primate hippocampus may undergo strong filtering by association areas (Lennert and Martinez-Trujillo 2013) and thus the primate “raw sensory cache” therefore does not have direct access to the high Hebbian plasticity circuits of the dentate gyrus, CA3 and CA1. The phylogenetic rerouting of hippocampal connectivity from early sensory to association areas in primates may be linked to the expansion of the primate neocortical mantle. One could speculate that with a more selective filter (i.e. a narrower connectivity), information would enter the hippocampus with heightened precision and congruence, thus exploiting the hippocampal Hebbian plasticity mechanisms to a greater extent, essentially ‘tuning it up’. This could be a mechanism for increasing the efficiency of memory formation in primate brains.

Studies in both rodents and humans have shown that the degree of long-term memory consolidation is strongly influenced by the affective significance of the event, involving interface with the amygdala (e.g. emotionally salient events; Cahill and McGaugh 1996), and the PFC (e.g. meaningful events, required by a task; Golby et al. 2001), with the PFC also being important for memory retrieval (Lepage et al. 2000). Long-term potentiation (LTP) in rodent CA3 is also greatly affected by neuromodulators such as norepinephrine (NE; Huang and Kandel 1996), which is also regulated by the amygdala (Valentino et al. 1998; Curtis et al. 2002), and the PFC (Arnsten and Goldman-Rakic 1984; Sara and Herve-Minvielle 1995; Jodo et al. 1998).

Some general organizational principles of the hippocampal system are similar in rodents and primates. However, it is noteworthy that the multisensory inputs into the hippocampal system in macaques retain a degree of segregation even at high levels of the cortical hierarchy (Fig. 4A). For example, the parahippocampal cortices TH and TF are associated with aspects of spatial processing, while the perirhinal cortices 35 and 36 (sometimes called TL) are associated with feature and object processing (Fig. 4A; Blatt et al. 2003; Suzuki and Amaral 2004; Bachevalier and Nemanic 2008). Thus, what appears to be a convergent system in wiring diagrams actually retains functional and connectional segregation with a finer-grained parcellation until very high levels.

However, despite some generally conserved principles, some critical species differences in hippocampal organization have emerged. For example, macaques have an uncal hippocampal extension absent in rodents (Rosene and Van Hoesen 1987; Barbas and Blatt 1995; Ding 2013), and species-specific connectional relationships, e.g. with amygdala (Fudge et al. 2012; Wang and Barbas 2018). In the reuniens nucleus of the thalamus, hippocampal afferents form specialized highly complex synaptic formations, reminiscent of synaptic triads formed by peripheral afferents to sensory nuclei (Joyce et al. 2022). Other emerging evidence suggests that there are some fundamental differences in the microstructural organization (Benavides-Piccione et al. 2020; Montero-Crespo et al. 2020) and neurochemical composition (Merino-Serrais et al. 2020; Tapia-González et al. 2020) of human CA1 that likely have significant consequences for local processing. Future studies will have to be performed to understand the full functional import of these differences.

### Functional segregation, parallel streams, and modularity

In addition to having a hippocampal system, larger-brained primates have a greatly expanded cortex, with increasing association cortices as a defining feature, as discussed above. Inter-areal connectomics show an expansion of segregated sensory processing streams (Ungerleider 1985; Kravitz et al. 2013), with frontal structures often emerging near the top (Fig. 4B). In macaques, dlPFC and anterior cingulate cortex (ACC) exhibit top–down functional roles and longer integration times for the processing of sensory stimuli (Goldman-Rakic 1987; Murray et al. 2014; Chaudhuri et al. 2015; Li and Wang 2022). In the marmoset, premotor areas emerge near the top in a recent hierarchical ranking (Theodoni et al. 2021). We will describe in a later section how top–down regulation of sensory streams (e.g. by the PFC) can gate information flow in coordination with relevant goals such as task demands. Working memory, abstraction, reasoning, and high-level decision-making all require the generation of highly specific but flexible mental representations in which information must often be deconstructed and recombined in both the processing of sensory events and in the reactivation of information stored in short- or long-term memory. For example, this deconstruction and reconstruction of information must occur in order to think about things we have never experienced, such as a pink elephant floating in the clouds singing Gershwin, as well as to extract dynamic rules and concepts from events around us (Wallis et al. 2001; Bunge et al. 2003). We propose that modular cortical organization as seen in primates is needed to support the generation and sustenance of the precise but dynamic mental representations needed for working memory, abstract reasoning, flexible decision-making, insights about oneself and others, and planning for the future. In support of this, modeling studies demonstrate that network modularity is necessary for working memory and abstraction (Kaiser and Hilgetag 2010; Rodriguez 2019; Changeux et al. 2021). These cognitive operations are particularly important to the survival of individuals, as well as communities, within complex social environments.

Recordings from the macaque dlPFC illustrate how these neurons can dynamically represent specific dimensions of sensory events that may not be possible if there was global, intersensory connectivity at early stages. For example, dlPFC neurons can generate and maintain the representation of highly specific categories of visual features (Freedman et al. 2001), with flexible, increased firing to the relevant dimension when seminal to the task (McKee et al. 2014). In contrast, neurons in inferior temporal cortex that process visual forms encode sensory information less dependent on task demands (McKee et al. 2014). This type of dynamic coding of a relevant dimension by the PFC can only occur if information is not “contaminated” with extraneous information at early stages in the sensory streams. Recent recordings show that the primate dlPFC can represent multiple, distinct information tracks in parallel (Roussy et al. 2022).

Relative sensory segregation may even persist as sensory streams reach the PFC in macaques (Fig. 4B). For example, the anatomical tracing studies of Goldman-Rakic (1987, 2011) demonstrated a segregation of sensory inputs to the PFC, resulting in an association of dorsal PFC with visual or auditory space, in contrast to the ventral PFC association with visual or auditory features (Fig. 4B). This is consonant with the synthesis of Ongür and Price (2000), where the lateral surface of the PFC generates and sustains representations of the outer world, and the ventral and medial surfaces represent the inner world, such as the viscera. A similar parallel organization can be seen with human fMRI (Haxby et al. 2000), although it important to note that both fMRI and physiological recordings can exhibit “reflected activity” from other areas, and only causal/lesion studies can demonstrate areas that are essential generators.

### Interactions between hippocampal and prefrontal systems in primates

Under optimal conditions, the convergent, multimodal, hippocampal memory system and the modular cortical system work together. However, there is little known at the cellular level about how this occurs in primates. Parts of the PFC and entorhinal cortex are reciprocally connected (Insausti et al. 1987; Muñoz and Insausti 2005), perhaps providing a dialogue with the hippocampus (this can also occur via the thalamus, e.g. Joyce et al. 2022). The hippocampus (CA1 and subiculum) projects directly to the PFC and most densely to the medial and orbital PFC (Barbas and Blatt 1995; Chiba et al. 2001; Price 2007; Aggleton et al. 2015). It is generally accepted that the PFC has no direct inputs to the hippocampal formation, although one early study reported that the dlPFC has direct projections to a posterior extension of the presubiculum near the anterior aspect of the calcarine sulcus (Goldman-Rakic et al. 1984), though this region can be challenging to distinguish from the ventral retrosplenial cortex, and its parcellation remains controversial (Kobayashi and Amaral 2000; Aggleton et al. 2015). The hippocampus is needed for working memory featuring delays longer than ~15 s (Zola-Morgan and Squire 1985) How the hippocampus and the PFC systems interact to sustain and manipulate information is an area for future research, though many indirect routes may exist to support this interaction (see discussion in Aggleton et al. 2015).

### Other structures that receive cortical convergence

Other structures receive convergent cortical input. One example is the striatum, where cortical areas that are otherwise connected with each other tend to project to the same striatal zones (Miller and Cohen 2001; Averbeck et al. 2014). This is particularly true for cortical areas that have been identified by cluster analyses to share common inputs (Giarrocco and Averbeck 2021, 2023). A second example is the thalamus, particularly association nuclei (e.g. the mediodorsal nucleus, or the pulvinar nucleus) which have expanded in primates, including humans (Baldwin et al. 2017; Chin et al. 2023; Giarrocco and Averbeck 2023). Although these pathways are not emphasized in this review, they also play a critical role in shaping all cortical processes. These structures have been implicated in a recent model of nested topological connectivity patterns among forebrain structures, based on parallel expansion between these structures during evolution, particularly with the addition of the dorsal pallium (Giarrocco and Averbeck 2023).

## The importance of top–down connectivity for controlling the contents of WM

A critical tool for abstract thought is the ability to employ top–down regulation of sensory inputs, and presumably of stored memories, to control the contents of the “mental sketch pad” according to current goals (also reviewed in Wang et al. 2020). Top–down gating of sensory processing over the early portions of sensory streams (Fig. 3) is essential to diminish processing of irrelevant distractions, promote focus on relevant dimensions, and activate and integrate specific dimensions (e.g. stored representations of pink elephants, the lyrics and melody of “Summertime,” etc.). In addition to extensive feedback projections from higher-order areas like the PFC, extensive local recurrent excitation within the PFC is necessary to generate and sustain the goals for top–down control, including powering long-range recurrent connections, notably with posterior association cortices (Levitt et al. 1993; Goldman-Rakic 1995; Chafee and Goldman-Rakic 2000; González-Burgos et al. 2000). This section will provide examples from primate lesions, physiology, and anatomy that demonstrate the importance of these top–down mechanisms emanating from the PFC.

It has been shown that PFC plays key role as driver of top–down control (Buschman and Miller 2007; Deco et al. 2023), with more abstract operations situated more rostrally (Nee and D'Esposito 2016). Top–down control has been most readily studied in terms of regulating attention to sensory events, where there is extensive evidence from studies of both humans and macaques. For example, humans with dlPFC lesions have impaired top–down control of attention, where they are unable to inhibit the processing of sensory distractors (Woods and Knight 1986; Knight et al. 1989; Yamaguchi and Knight 1990; Chao and Knight 1995), and are also less capable of strengthening the processing of relevant sensory stimuli (Barceló et al. 2000). Similar findings have been seen in macaques, where dlPFC lesions make animals more susceptible to interference during working memory (Bartus and Levere 1977), and physiological recordings show that dlPFC is involved with top–down control of attention (Buschman and Miller 2007; Panichello and Buschman 2021), including projections from the frontal eye field back to V4 to gate early visual responses in coordination with eye movements (Moore and Armstrong 2003).

Physiological recordings coupled with reversible lesions have helped to establish a causal role for the macaque dlPFC in top–down control of attention. A classic study by Fuster showed that cooling the dlPFC reduced the firing of inferior temporal cortex (ITC) neurons to the correct color, and that this was particularly evident in neurons in superficial layers of ITC (Fuster et al. 1985). Consistent with this physiology, lesioning of the dlPFC impaired performance on a related task when top–down control was needed for performance (Rossi et al. 2007). Recordings from the dlPFC during visuospatial working memory show that dlPFC “delay cells” that represent a location in visual space during working memory are resistant to distractors, in contrast to neurons in parietal association cortex area LIP (Carlson et al. 1997; Suzuki and Gottlieb 2013) and that reversible inactivation of the dlPFC produced much larger increases in distractibility than inactivation of LIP (Suzuki and Gottlieb 2013), a finding replicated by computational models in which the PFC has stronger recurrent connectivity than LIP (Murray et al. 2017; Froudist-Walsh et al. 2021). The ability of the dlPFC to maintain persistent firing even in the presence of distractors develops with age (Zhou et al. 2013), consistent with the late maturation of the PFC in primates (Rakic et al. 1994).

“Top–down” influences can even be seen extending to macaque V1. V1 receives feedback from many of the same visual areas to which it projects, such as V2, V3, V4, and MT, but receives almost no direct projections from the PFC (Xu et al. 2022) with the exception of area 8l (Markov et al. 2014a). Supporting this view, V1 shows only small “top–down” modulations during attention or working memory tasks (Yoshor et al. 2007; Leavitt et al. 2017; Martinez-Trujillo 2022). This view would be consistent with keeping a relatively “raw data” cache in V1, and filtering information based on top–down goals at higher levels of the hierarchy.

### Anatomy of top–down modulation in primates

As previously described, the term “top–down” is often associated with functional studies demonstrating that association areas can tune information in sensory areas (Treue and Maunsell 1996; McAdams and Maunsell 1999; Berman and Colby 2009). Anatomical studies using neural tracers have suggested possible circuit mechanisms for the modification of information in sensory streams via cortico-cortical (Medalla and Barbas 2014), cortico-thalamic (e.g. Zikopoulos and Barbas 2012), and cortico-striatal projections (e.g. Choi et al. 2017), which may be substrates for top–down regulation such as attention. Here, we describe examples of cortico-cortical and cortico-thalamic connectional architecture that may subserve such functions. Cortico-cortical “top–down” connections, particularly in sensory streams, often take the form of feedback patterns of laminar innervation. Although these connection patterns are often more complex than modeled, a deeper study of some of these feedback connections provides clues as to how PFC projections can shape information flow in sensory streams. Here, we will highlight the few studies examining in fine detail the frontal eye field projections to visual association cortices (Anderson et al. 2011) and projections from the frontal polar area 10 to the superior temporal gyrus (STG; reviewed in: Medalla and Barbas 2014); note that there are currently no studies examining the feedback projections from the dlPFC anterior to the FEF at this level of detail. In all cases, prefrontal projections predominantly target the spines of putative excitatory neurons, with a much smaller, but likely of great functional import, proportion innervating putative inhibitory neurons. Quantifying the inhibitory targets of long-range projections may be particularly valuable for understanding how specialization of function may emerge in the densely connected mouse cortex. Indeed, although mesoscopic connectivity can be used to predict patterns of working memory activity in the monkey cortex (Froudist-Walsh et al. 2021; Mejías and Wang 2022), in the mouse such predictions require knowledge of the cell-type targets (Ding et al. 2024).

One study used electron microscopy to examine the termination patterns of the frontal eye fields (FEF, area 8) in V4 and lateral intraparietal cortex (LIP; Anderson et al. 2011). It should be noted that the frontal eye field is a motor area, whose connections thus may have properties specific to coordinating eye movements with visual processing. Though this study did not quantify the laminar distribution of axon terminations, other studies have demonstrated that subdivisions of the FEF can have distinct feedforward and feedback laminar termination patterns in subdivisions of LIP, which can be correlated with gradients in neuronal density of the origin and termination cortices (Medalla and Barbas 2006), a demonstration of the complexity of connectional networks, the benefit of small injection sites, and an illustration that prefrontal projections to sensory streams are not exclusively feedback. In both V4 and LIP, FEF terminations across layers targeted spines and a variable amount of putatively inhibitory dendritic shafts, with higher levels of spine innervation in superficial layers (V4: 85% to 92% spines; LIP 78% to 93% spines). The V4 to V2 projection, a feedback projection occurring earlier in the dorsal stream, mostly targets layer I with minor termination zones in II to III and VI, and targeted spines at a lower frequency (~80%; Anderson and Martin 2006).

These studies in the dorsal visual stream indicate that prefrontal projections likely gain access to local circuits mainly through contact with pyramidal neurons, and a small cohort of synapses formed on inhibitory neurons. Though the authors were not able to discern the types of inhibitory neurons that were targeted by these projections, inhibitory neuron class likely plays an important role in the impact of afferents on local circuits (Wang et al. 2004)*.* In macaques, Torres-Gomez et al. (2020) have demonstrated varying proportions of inhibitory neuron types in superficial layers of a selection of cortices along the dorsal stream, ranging from early visual areas to the dlPFC. Though their study did not explicitly include V4 or LIP, the range of cortices covered overlaps with these areas along the dorsal stream. In MT, parvalbumin (PV) positive inhibitory neurons were the most frequent, while in dlPFC, calretinin (CR) positive inhibitory neurons were the most frequent. These two types of inhibitory neurons display different innervation patterns within the cortical circuit, so their recruitment by afferents from other areas likely introduces disparate consequences for local activity. It is still unclear which inhibitory neurons are targeted by incoming afferents in each of these areas. Notably absent from the literature are studies examining the visual stream postsynaptic targets originating from more anterior parts of the dlPFC, like areas 46 and 9, known for their role in working memory and executive function. This type of information will be an important contribution given that the dorsal stream is one of the most studied physiological systems in nonhuman primates.

**Fig. 5:**
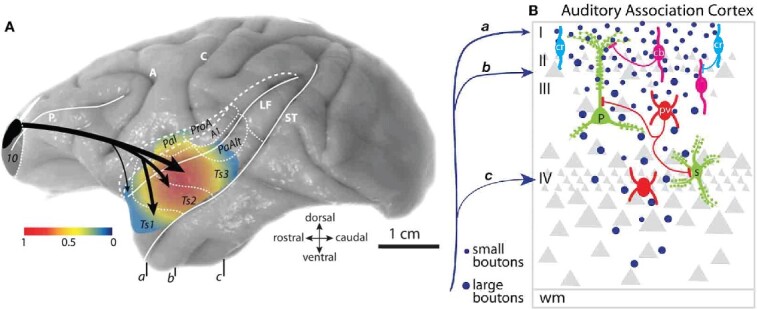
Frontopolar- “top-down” frontopolar projections to auditory association areas of the superior temporal gyrus. Area 10 axons terminate in a feedback pattern, with smaller frequent boutons in layer I and larger, sparser boutons in mid-to-deep layers. Terminations in layer I likely interact with disinhibitory inhibitory neurons (CR, calretinin), while in layer II to III, they interact more frequently with inhibitory neurons that target the apical dendrites (CB, calbindin) or pyramidal neurons; in mid-deep layers, they are more likely to target perisomatic inhibitory neurons (PV, parvalbumin). Adapted from Figs 4 and 5 of Medalla and Barbas (2014).

The feedback projections from frontal polar area 10 to the STG auditory association areas Ts1, Ts2, and Ts3 have been examined in a set of studies quantifying not only their laminar termination patterns, bouton characteristics, and postsynaptic targets with excitatory neurons, but importantly also their synaptic interactions with different classes of inhibitory neurons, demonstrating with finer detail how the PFC can influence sensory processing within auditory streams (Fig. 5). Terminations coming from area 10 predominate in superficial layers of the STG (79% to 87%), demonstrating a strong feedback termination pattern for this projection (Barbas et al. 2005). The authors also found a progressive increase in the size of area 10 axon terminals from layer I to layer IV (Germuska et al. 2006; Medalla et al. 2007; Medalla and Barbas 2014), suggesting that though fewer, when present in layer IV, area 10 boutons may have a larger synaptic effect on their targets (Germuska et al. 2006). More frequent, but smaller boutons in layer I may provide a low tonic tone of excitation for postsynaptic targets, including inhibitory neurons. In contrast to feedforward projections that target the middle layers of a cortical column, which tend to be more focally concentrated, and which may provide a strong net excitatory effect, feedback projections, which have a wider breadth in termination zone particularly in layer I, may have more diverse net effects cortical microcircuitry (Rockland and Virga 1989; Javadzadeh and Hofer 2022).

The inhibitory microsystem in superficial layers of STG likely affects how area 10 signals gain entry into local circuitry. Like prefrontal projections to the dorsal visual stream, the majority of area 10 terminations targeted the spines of putative excitatory neurons in STG, while the remainder targeted functionally distinct inhibitory neurons dependent on the layer. In primates, labeling of inhibitory neurons is often performed using the calcium-binding proteins calbindin (CB), PV, and CR (Torres-Gomez et al. 2020), because these markers are largely nonoverlapping in primates, label 90% + percent of inhibitory neurons in primate cortex, and feature some useful functional distinctions (Condé et al. 1994; DeFelipe 1997; Wang et al. 2004; Medalla et al. 2023). In STG, layers II-III are populated in highest number by CR neurons, followed by CB neurons, and then PV neurons (Kondo et al. 1999). In superficial layers of primate cortex, CR neurons are thought to have a predominantly disinhibitory role via inhibition of other inhibitory neurons (DeFelipe 1997; Meskenaite 1997; Wang et al. 2004; Melchitzky et al. 2005), while CB neurons target the dendritic tree of pyramidal apical dendrites, and PV neurons have a perisomatic targeting pattern on pyramidal neurons (DeFelipe 1997). The authors note more area 10 termination contacts in layers II to III with CB neurons than PV neurons, and while they did not label CR neurons, all contacts with inhibitory neurons in layer I were CB and PV negative, suggesting the bulk of inhibitory targets were CR positive (Medalla et al. 2007). Contacts with CB neurons may sculpt activity in the apical dendritic trees of pyramidal neurons, perhaps decreasing noisy or irrelevant inputs and gating sensory inputs to the working memory network (Froudist-Walsh et al. 2021). Area 10 contacts with CR neurons in layer I may play another important “top–down” role, namely, in the permissive routing or gating of incoming signals, as suggested by modeling (Yang et al. 2016) and physiological studies (Krabbe et al. 2019). This may be a mechanism for top–down disinhibition-mediated amplification of pertinent incoming signals. Disinhibition-mediated gating may be an important component of primate specialization, as the increase in inhibitory neurons seen in primate cortices is driven by an expansion of the CR class (Džaja et al. 2014). Area 10 terminations may thus initiate local circuit mechanisms to highlight pertinent signals in a “top–down” fashion via CR-mediated disinhibition (Yang et al. 2016), while closing the gate on distracting information via CB-mediated inhibition (Wang et al. 2004; Froudist-Walsh et al. 2021). As CB and CR neurons likely express distinct neuromodulatory receptors (Tremblay et al. 2016), the mix and levels of circulating neuromodulators in cortex (here STG) may determine whether inhibitory or disinhibitory feedback mechanisms dominate at a particular time.

Another opportunity for top–down modulation early in sensory streams occurs via specialized macaque projections to the thalamic reticular nucleus (TRN). The TRN is an entirely inhibitory nucleus that gates output from other thalamic nuclei. The TRN receives afferents from corticothalamic and thalamocortical projections, but it is positioned to dampen thalamocortical output (Zikopoulos and Barbas 2007). Sensory cortices and their thalamic counterparts interact with topographic TRN sectors, but elements of the macaque prefrontal cortex interact with the breadth of the TRN, outside the prefrontal sector (Zikopoulos and Barbas 2006). One example is the dlPFC, which does not have a rodent homologue. Thus, the macaque PFC is specialized to recruit the TRN to gate thalamic output in other modalities. This may be an additional mechanism for top–down modification of activity in sensory streams and could play an important role in estimating decision confidence (Jaramillo et al. 2019).

## The evolution of molecular mechanisms to regulate contents of WM

**Fig. 6:**
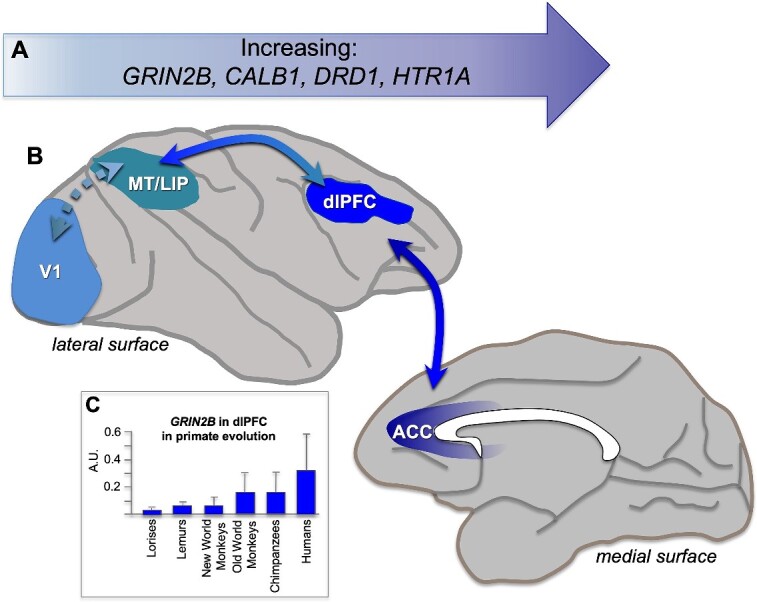
Molecular gradients aligned with the cortical hierarchy in primates. (A) As described in the text, research has shown increasing expression of *GRIN2B, CALB1, DRD1*, and *HTR1A* encoding the GluN2B subunit of the NMDAR that closes slowly and fluxes high levels of calcium, the calcium-binding protein, calbindin, the dopamine D1 receptor, and the serotonin 5HT_1A_ receptor, respectively. GRIN2B (NMDAR-GluN2B) are essential to persistent dlPFC neuronal firing during working memory. (B) The cortical hierarchy across the dorsal stream with increasing timescales across regions, from V1 to MT, to LIP to dlPFC to ACC (anterior cingulate), areas often used to compare molecular expression levels. (C) The levels of *GRIN2B* expression in dlPFC increase across primate brain evolution, adapted from Muntané et al. (2015). Figure based on Murray et al. (2014); Burt et al. (2018); Arnsten et al. (2021); Froudist-Walsh et al. (2021); Froudist-Walsh et al. (2023).

The contents of working memory are further refined by neuromodulatory influences, enhancing the coordination of cognitive state with arousal state (Arnsten et al. 2012). Studies of macaque and human cortex show positive correlations between intrinsic timescale and increasing *GRIN2B* (NMDAR-GluN2B) and *CALB1* (the calcium buffering protein CB) expression across the cortical hierarchy, consistent with increasing integration and persistent firing requiring higher levels of calcium at higher levels of the hierarchy (Fig. 6A and B; Murray et al. 2014; Burt et al. 2018; Arnsten et al. 2021). Indeed, there is an evolutionary increase in *GRIN2B* expression in the primate dlPFC (Fig. 6C), suggesting the increasing use of this receptor with the expansion of abstract cognition. There are also gradients in neuromodulatory receptors (Fig. 6A and B), including an increase in the density of the dopamine (DA) D1 receptor (D1R; Froudist-Walsh et al. 2021). However, we still need to understand how these gradients translate to cell-specific expression, as many studies utilized tissue homogenates or otherwise did not distinguish cell type.

**Fig. 7:**
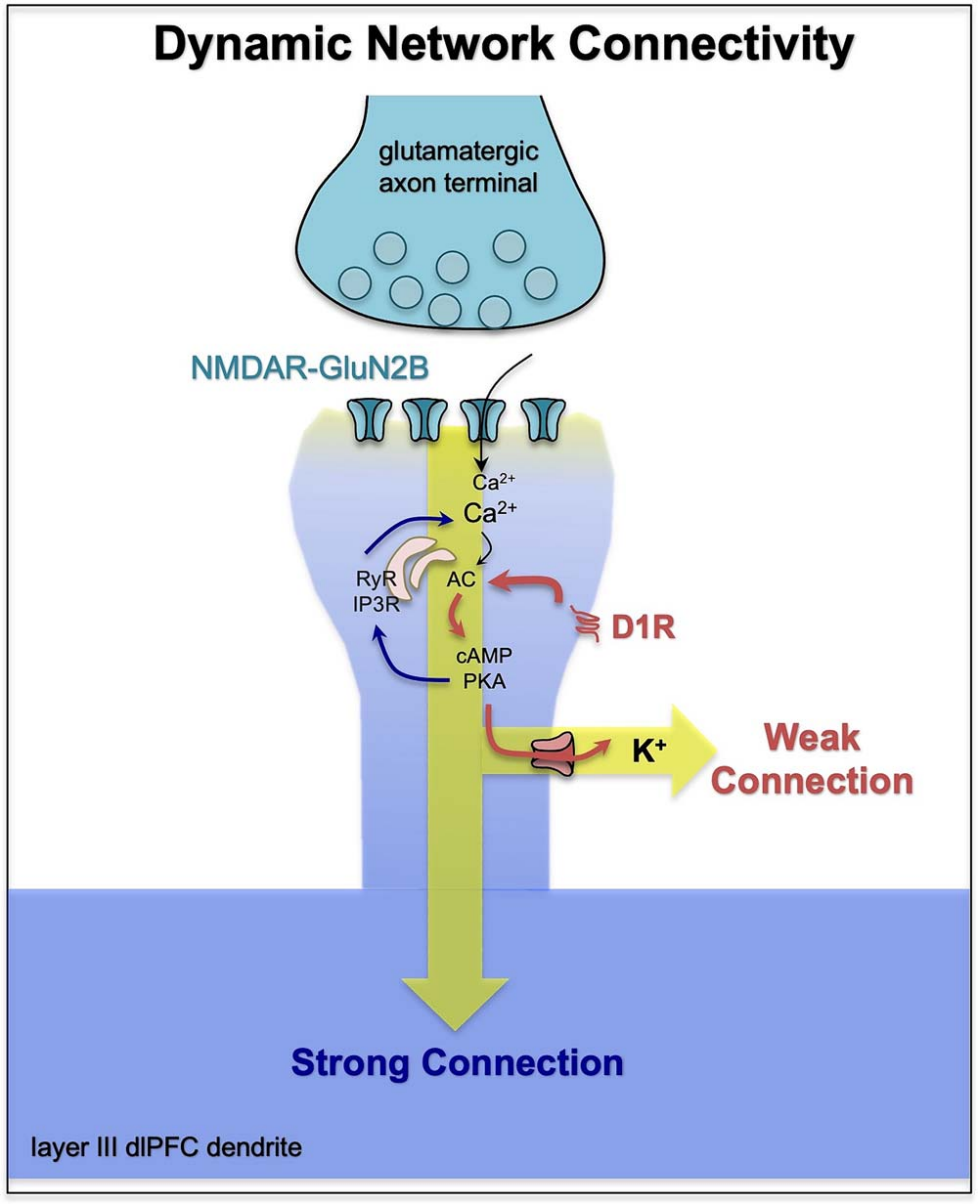
A working model of how dopamine actions at D1R in layer III of dlPFC may sculpt network inputs and refine the contents of working memory. Dopamine D1R are concentrated on dendritic spines in layer III dlPFC, often at extrasynaptic locations where they are colocalized with ion channels that weaken network connectivity, such as HCN-slack channels (Paspalas et al. 2013; Gamo et al. 2015; Wu et al. 2024). Thus, weaker (nonpreferred) inputs may be differentially gated out. Enhancement of lateral inhibition from PV interneurons may also contribute to the refinement of representations in working memory. Based on Vijayraghavan et al. (2007) and Arnsten et al. (2021).

The effects of DA at D1R have received particular focus, where decades of research have shown these actions to be essential to dlPFC operations (Brozoski et al. 1979; Sawaguchi and Goldman-Rakic 1994; Vijayraghavan et al. 2007; Puig and Miller 2012; Vijayraghavan et al. 2016; Wang et al. 2019). Particularly relevant to the current discussion is the finding that optimal levels of DA D1R stimulation in dlPFC can “sculpt” the tuning of delay cells, by decreasing responses to nonpreferred stimuli (Vijayraghavan et al. 2007; Fig. 7). As there is increased DA release in dlPFC to salient events (Kodama et al. 2014), this may be an additional mechanism by which the contents of working memory can be tuned in accordance with arousal state. Dopamine release in the cortex may therefore act as a signal that salient or reward-predicting stimuli should be protected in working memory and make it more difficult for future sensory stimuli to disrupt this representation (Froudist-Walsh et al. 2021). With high levels of catecholamine release as occurs during uncontrollable stress, the dlPFC is fully disconnected, with loss of delay cell firing, switching control of behavior to other circuits (Arnsten 2015). Thus, these neuromodulatory actions may act in combination with top–down projections to refine the contents of working memory, and to orchestrate brain state in response to the environment. More generally, neuromodulation may enable considerable flexibility in the gating of different information into cortical networks by flexibly enhancing or shutting off excitatory signaling at dendritic spines and inhibition via distinct cell types. This flexible control of connectivity pathways has been termed dynamic network connectivity (Arnsten et al. 2010; Arnsten et al. 2012).

However, neuromodulation and dynamic network connectivity do not affect the whole cortex equally. The density of most neuromodulatory receptor types per neuron increases systematically along a gradient that aligns with the cortical hierarchy in the macaque (Froudist-Walsh et al. 2023; Fig. 6). Of all 14 studied receptor types, the dopamine D1 receptor expression has the strongest positive correlation with the cortical hierarchy (Froudist-Walsh et al. 2021). The gradient in expression of DA and D1R across the cortical hierarchy suggests that these modulatory mechanisms are less prevalent in V1 and increase across the visual streams into the PFC (Froudist-Walsh et al. 2021). Indeed, the available anatomical and physiological evidence suggests that, in contrast to the dlPFC, macaque V1 may be less influenced by neuromodulators (Froudist-Walsh et al. 2023), consistent with it providing a more faithful cache of “raw data.” Thus, the DA innervation of macaque V1 is limited to layer I (Berger et al. 1988), with a sparse noradrenergic innervation as well (Kosofsky et al. 1984; Levitt et al. 1984; Morrison and Foote 1986). These anatomical data are consistent with physiological studies showing that local application of DA into macaque V1 has little effect on V1 neuronal firing (Zaldivar et al. 2014).

Although there appear to be little effects of DA in primate V1, cholinergic inputs may have greater influences than the catecholamines (Coppola and Disney 2018; Krueger and Disney 2019). Muscarinic receptors are concentrated on GABAergic interneurons in macaque V1, and local application of acetylcholine reduces V1 neuronal firing (Disney et al. 2012). Indeed, muscarinic M2 receptors are an excellent, evolutionarily conserved marker of primary sensory cortex (Zilles and Palomero-Gallagher 2017). Interestingly, cortical areas at higher levels of the cortical hierarchy have more extensive muscarinic receptor expression on pyramidal cells (Disney et al. 2014), consistent with the general theme of greater neuromodulatory influences at higher levels of the cortical hierarchy. There is also a relatively rich serotonergic innervation of macaque V1 (Kosofsky et al. 1984; Levitt et al. 1984; Morrison and Foote 1986), as well as high expression of serotonergic 5HT2 receptors (Lidow et al. 1989; Zilles and Palomero-Gallagher 2017), but the influence of serotonin receptor mechanisms on V1 neuronal firing is currently unknown. Little is known about serotonin actions in the macaque dlPFC (Williams et al. 2002), and these will be important areas for future research. The most unique pattern of expression of all receptors in the primate brain is that of the serotonin 5-HT1A receptor, which increases 17-fold from V1 to subgenual and anterior cingulate (Froudist-Walsh et al. 2023; Fig. 6). Given that neuron density decreases by almost 5-fold from V1 to frontal and cingulate cortex (Collins et al. 2010), this enables much greater capacity for neuromodulation of single neurons and dynamic network connectivity in the primate prefrontal and cingulate cortex. A similar pattern of serotonin 5-HT1A receptor expression exists in the human and rat brain, although the gradient of expression is noticeably flatter in the rat cortex (Froudist-Walsh et al. 2023). Thus, in addition to a flattened connectivity hierarchy (see above), the rodent brain may also have a flattened receptor gradient. This could help explain findings that rodents’ primary sensory cortex is more strongly modulated by state than the primate V1. At the other end of the hierarchy, these results also intriguingly point to greater capacity for flexible routing of information near the top of the hierarchy in the primate brain compared to that of the rodent.

## Summary

In summary, emerging analyses of cortical areal connectivity across species have revealed not only some similarities but also many marked differences between rodent and primate cortical organization. The convergence of connections into the hippocampus has been a highly successful strategy for the creation of episodic memories, conserved across species, while larger, primate cortices have added computational space for modular processing essential to abstraction and working memory. Although the reduction in density of cortical connectivity in primates has sometimes been considered a necessary sacrifice to constrain brain size within the skull, we argue that the modular nature of long-range connections in primate, in combination with top–down regulation from higher areas in the cortical hierarchy, are essential to the deconstruction and reconstruction of information central to abstract thought. Given the pervasive use of mouse models in neuroscience research, these fundamental differences in cortical organization between rodents and primates must be appreciated if we are to effectively translate across species.
